# Community pharmacy services to optimise the use of medications for mental illness: a systematic review

**DOI:** 10.1186/1743-8462-2-29

**Published:** 2005-12-07

**Authors:** Simon Bell, Andrew J McLachlan, Parisa Aslani, Paula Whitehead, Timothy F Chen

**Affiliations:** 1 Faculty of Pharmacy, The University of Sydney, New South Wales 2006, Australia; 2 School of Pharmacy, Curtin University of Technology, Bentley, Western Australia 6102, Australia

## Abstract

The objective of this systematic review was to evaluate the impact of pharmacist delivered community-based services to optimise the use of medications for mental illness. Twenty-two controlled (randomised and non-randomised) studies of pharmacists' interventions in community and residential aged care settings identified in international scientific literature were included for review. Papers were assessed for study design, service recipient, country of origin, intervention type, number of participating pharmacists, methodological quality and outcome measurement. Three studies showed that pharmacists' medication counselling and treatment monitoring can improve adherence to antidepressant medications among those commencing treatment when calculated using an intention-to-treat analysis. Four trials demonstrated that pharmacist conducted medication reviews may reduce the number of potentially inappropriate medications prescribed to those at high risk of medication misadventure. The results of this review provide some evidence that pharmacists can contribute to optimising the use of medications for mental illness in the community setting. However, more well designed studies are needed to assess the impact of pharmacists as members of community mental health teams and as providers of comprehensive medicines information to people with schizophrenia and bipolar disorder

## Introduction

Mental and behavioural disorders are estimated to account for 12% of the global burden of disease [1]. More than 450 million people worldwide suffer from a diagnosable mental illness, and four of the six leading causes of years lived with disability are due to neuropsychiatric disorders [1]. Much of the burden of mental illness is managed in the community setting. In 2003–04 mental health related medications accounted for 10.9% (17.8 million) of all medications prescribed by general medical practitioners in Australia [2]. Although community care offers many advantages over institutional care, community care can place extra demands on family, friends and primary health practitioners [3]. Health professionals have identified people with mental illness as among their most challenging patients to manage [4]. Improving the quality and accessibility of community care for people with mental illnesses is an aim outlined in the parliamentary report *Mental Health Services in New South Wales *[5].

The appropriate use of medications is central to the effective management of mental illnesses, however, there is evidence that psychotropic medications are often used inappropriately [6,7]. Elderly people are especially sensitive to the effects of psychotropic medications, and may be susceptible to adverse reactions including cardiac toxicity, confusion and unwanted sedation [8]. Psychosocial problems, the emergence of side effects, and the delayed onset of action of anti-depressant medications, may be contributing factors in high rates of medication non-adherence [9,10]. Medical co-morbidity is also common, and polypharmacy increases the risk of drug-drug interactions and medication misadventure [11].

The World Health Organization (WHO) has recognised including pharmacists as active members of the health care team as one approach to improving psychotropic medication use [6]. The National Strategy for the Quality Use of Medicines in Australia highlights the importance of a multidisciplinary approach to improving medication use [12]. The development of new roles for pharmacists has expanded the opportunities for pharmacists to provide community-based services to users of psychotropic medications. The Third Community Pharmacy Agreement, signed between the Australian Government and Pharmacy Guild of Australia in 2000, provided remuneration for pharmacists in Australia to conduct medication management reviews in the community setting (referred to as 'Home Medicines Review') and to provide consumer medicine information (CMI) [13]. Residential medication management reviews, initially funded through the Second Community Pharmacy Agreement in 1995, are available to all permanent residents of accredited aged care facilities in Australia [14]. A systematic review of the role of pharmacists in mental health care, published in 2003, concluded that pharmacists can bring about improvements in the safe and efficacious use of psychotropic medications [15]. The review included seven studies conducted for hospital inpatients and nine studies conducted in residential aged care or outpatient settings. Since that time pharmacists and pharmacy practice researchers have developed additional community pharmacy services in speciality areas. This has corresponded with a significant increase in the volume of published research on community-based services provided by pharmacists relating to mental health. The objective of this systematic review was to specifically evaluate the impact of pharmacist delivered *community-based *services to optimise the use of medications for mental illness.

## Methods

### Literature search strategy

Medline (1966-April 2005), Embase (1994-April 2005), PsychInfo (1985-April 2005), Cinahl (1982-April 2005), International Pharmaceutical Abstracts (1970-April 2005) and the Cochrane Controlled Trials Register (2^nd ^quarter 2005) were searched using text words and MeSH headings including: *pharmacy, pharmacists, pharmaceutical care*, *pharmaceutical services, mental disorders, mentally ill persons, depression, schizophrenia, psychotic disorders, antidepressive agents, psychotropic drugs, benzodiazepines, anxiety and antipsychotic agents*. Reference lists of retrieved articles were checked for additional studies not identified in the original database search. If the abstract clearly indicated that the study did not relate to pharmaceutical services provided by pharmacists to optimise the use of medications for mental illness, or if the study was conducted in an acute inpatient or hospital setting, then the study was excluded at this stage.

### Inclusion criteria and review procedure

**Figure 1:**
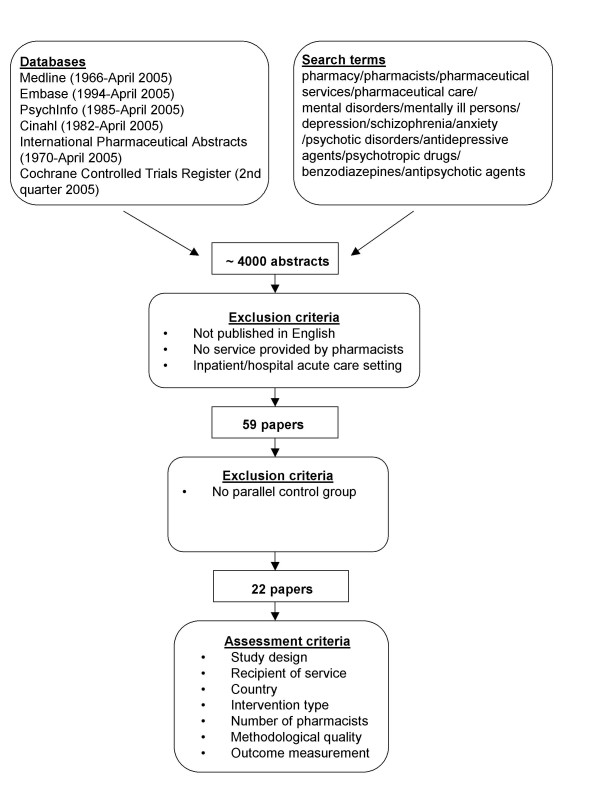
Literature search strategy and review procedure.

Studies published in English, with a parallel control group (randomised and non-randomised) that reported the provision of services by pharmacists in community and residential aged care settings were considered. This included trials specifically conducted for individuals with a mental illness, or that reported outcomes in terms of changes to mental health symptoms, and studies of medication reviews and education initiatives to optimise the use of medications commonly prescribed for mental illness. Papers that reported pharmacists' activities as part of multidisciplinary teams were included where a pharmacist or pharmacists provided a service specifically related to optimising the use of medications for mental illness. Studies of pharmacists' interventions in residential aged care facilities were included, because community pharmacists frequently provide services to residential aged care facilities, but studies evaluating pharmacists' services in hospital inpatient or acute care settings were excluded. Studies without control groups, before and after studies, descriptive studies, results of postal surveys and qualitative interviews were excluded, as were studies to optimise medication use that did not involve a service provided by pharmacists. Each study meeting the criteria outlined above was assessed on the basis of study design, service recipient, country of origin, intervention type, number of participating pharmacists, methodological quality and outcome measurement. An overview of the literature search strategy and review procedure is presented in Figure 1.

## Results

The literature search identified 59 papers that reported or discussed community pharmacy services to optimise the use of medications for mental illness. Twenty-two papers reported the results of studies that met the inclusion criteria for the review. Studies that met the inclusion criteria were approximately equally divided between services provided to consumers (n = 10) (Table 1), and services provided to other health care professionals (n = 12) (Table 2). All but one included study were conducted in developed countries, and 15 of the 22 papers were published in the last six years. Thirty-seven papers were excluded from the review for the following reasons. Thirteen papers reported data from descriptive studies [16-28] and nine papers reported outcomes of qualitative interviews or focus groups [29-37]. Five papers reported results of before and after interventions or were cohort studies without parallel control groups [38-42]. Six papers reported results of postal surveys [43-48]. Three papers presented study methods only [49-51], and one study was conducted by pharmacy researchers but did not report the outcomes of a service provided by pharmacists [52].

**Table 1:** Services provided to consumers

**Reference**	**Country**	**Design**	**Setting**	**Service**	**No. Ph**	**Sample size**	**Main outcome measures**	**Significant outcomes**
Brook et al, (2003)	NL	RCT	CP	PE	19	64 Int 71 Cont	Attitudes to AD	Improved attitudes
Brook et al, (2003)	NL	RCT	CP	PE	19	64 Int 71 Cont	Depressive symptoms	Improvements in depressive symptoms (significance dependent on treatment of missing data)
Brook et al, (2005)	NL	RCT	CP	PE	19	64 Int 71 Cont	AD adherence, depressive symptoms	Improved adherence among those that completed pharmacist intervention. Intention to treat analysis no difference.
Finley et al, (2002)	USA	CT	HMO	PE/TM	2	91 Int 129 Cont	AD adherence, resource utilisation, depressive symptoms, medication switch rates, patient satisfaction.	Improved adherence, higher medication switch rates, decline in patient visits to primary care providers, improved patient satisfaction
Finely et al, (2003)	USA	RCT	HMO	PE/TM	2	75 Int 50 Cont	AD adherence, resource utilisation, depressive symptoms, patient satisfaction, medication costs.	Improved adherence, improved patient satisfaction
Capoccia et al, (2004)	USA	RCT	PCM	PE/TM	2	41 Int 33 Cont	AD adherence, resource utilisation, depressive symptoms, quality of life, patient satisfaction.	
Adler et al, (2004)	USA	RCT	PCM	PE/TM	5	268 Int 265 Cont	AD use rates, depressive symptoms.	Improved AD use rate.
Rosen et al, (1978)	USA	CT	CMH	PE/TM	1	30 Int 152 Cont	Patient well-being, patient satisfaction, quantity and cost of service provision.	Intervention patients' had higher personal adjustment scores, were 'better since coming to clinic' and less likely to need further help.
Razali et al, (1995)	Malaysia	RCT	OP	PE	1	85 Int 80 Cont	Relapses requiring hospital readmission.	Fewer relapses requiring hospitalisation in intervention group.
Shaw et al, (2000)	UK	RCT	OP	PE/CA	1	51 Int 46 Cont	Medication knowledge, medication related problems, adherence, hospital readmission.	

RCT = randomised controlled trial; CT = controlled trial; CP = community pharmacy; HMO = health maintenance organisation; PCM = primary care medical centre; CMH = community mental health centre; OP = outpatients' clinic; NL= The Netherlands; PE = patient education; TM = treatment monitoring; ca = care planning; Ph = pharmacists; Int = intervention group; Cont = control group; AD = antidepressant medication.

**Table 2:** Services provided to other health professionals

**Reference**	**Country**	**Design**	**Setting**	**Service**	**No. Ph**	**Sample size**	**Main outcome measures**	**Significant Outcomes**
Williams et al, (2004)	USA	RCT	GP	MR	1	63 Int 77 Cont	Physical, cognitive and affective functioning, health status, number and cost of medications.	Decrease in number and cost of medications
Schmidt et al, (1998)	Sweden	CRCT	RAC	MR	15	626 Int 1228 Cont	Incidence and quality of psychotropic medication use.	Increase in psychotropic medication use and therapeutic duplication in control group. Decrease in antipsychotic and hypnotic use in intervention group, decrease in AD use in intervention and control groups
Schmidt et al, (2000)	Sweden	CRCT	RAC	MR	15	1549 Total ^†^	Quality of medication use (three-year follow-up).	Previous Improvements in quality of medication use sustained for specific indicators
Coleman et al, (1999)	USA	CRCT	HMO	MR	1	96 Int 73 Cont	Depressive symptoms, physical function, service utilisation, number of high risk medications, satisfaction, urinary incontinence, falls, cost.	Decrease in urinary incontinence in intervention group at 12 months. No differences between intervention and control groups at 24 months
Roberts et al, (2001)	Australia	CRCT	RAC	MR	ND	905 Int 2325 Cont	Medication use, medication cost, mortality, morbidity and resource utilisation.	Decrease in psycholeptic and benzodiazepine drug administration, decrease medication cost
Furniss et al, (2000)	UK	CRCT	RAC	MR	1	158 Int 172 Cont	Prescribing patterns, mortality, mental state, depressive symptoms, and behavioural disturbance	Decrease in mortality, decrease in number of prescribed medications, increase in behavioural disturbance
Burns et al, (2000)	UK	CRCT	RAC	MR	1	158 Int 177 Cont	Medication costs.	Decrease in medication cost
van Eijk et al, (2001)	NL	CRCT	GP	ED	37^‡^	70 Int 1* 52 Int 2* 68 Cont*	Prescribing of highly anticholinergic and less anticholinergic AD medications	Decrease in high anticholinergic AD use in intervention group 2. Increase in less anticholinergic AD use in Intervention group 1
Hartlaub et al, (1993)	USA	CT	PPGP	ED	ND	28 Int 1* 26 Int 2* 37 Cont *	Benzodiazepine prescribing pattern	
de Burgh et al, (1995)	Australia	RCT	GP	ED	1	142 Int * 144 Cont *	Benzodiazepine prescribing pattern	Overall decline in benzodiazepine use. Differences between intervention and control groups not significant
Crotty et al, (2004)	Australia	CRCT	RAC	ED	1	381 Int 334 Cont	Fall rate, psychotropic medication use, BP, quality of life	Increase in 'as required' antipsychotic medication use in the intervention group
Avorn et al, (1992)	USA	CRCT	RAC	ED	1	431 Int 392 Cont	Psychotropic mediation use, mental status, memory, anxiety, depressive symptoms, behaviour, sleep	Decrease in psychotropic medication use, decrease in inappropriateness of drug use, less cognitive decline, increase in depression scores.

CT = controlled trial; RCT = randomised controlled trial; CRCT = cluster randomised controlled trial; GP = general practice; RAC = residential aged care; PPGP = prepaid group practice; HMO = health maintenance organisation; USA = United States of America; NL = The Netherlands; MR = medication review; ED = prescribing education initiative; ND = not described in paper; Int = intervention group; Cont = control group; AD = antidepressant medication.

† Journal article reported overall number of patients (n = 1549) divided between 16 intervention and 18 control residential aged care facilities.

‡ Pharmacists participated in group discussions with physicians, discussions were led by a medical researcher.

* Reported sample size based on number of physicians that received pharmacists' educational intervention.

Papers that met the review inclusion criteria reported the outcomes of medication counselling by community pharmacists at the time of dispensing, education and monitoring activities conducted at primary care medical centres and staff model health maintenance organisations (HMOs), discharge medication counselling, and medication monitoring at a community mental health centre. Pharmacist delivered services provided to other health professionals included medication reviews and outreach education activities designed to optimise prescribing. Several medication review studies reported impacts of pharmacists' interventions in terms of changes in prescribing of medications commonly used to treat mental illness and/or changes in mental health symptoms, but were not specifically targeted to people with a mental illness. Several small studies of pharmacists' medication review activities specifically conducted for people with a mental illness did not meet the review inclusion criteria [17,18,20,22,41,42].

### Services provided in community pharmacies

Three papers reported results of community pharmacists' medication counselling sessions for people commencing non-tricyclic antidepressant therapy in The Netherlands [53-55]. Intervention patients participated in three counselling sessions (lasting between 10 and 20 minutes each) and received a take-home video that emphasised the importance of medication adherence. The medication counselling sessions involved pharmacists informing patients about the appropriate use of their medication. This included providing information about the benefits of taking the medication, informing patients about potential side-effects, informing patients about the onset of action for antidepressant medications and reinforcing the need for patients to take their medication on a daily basis. At three month follow-up the intervention patients had significantly more positive drug attitudes than controls [53], and at six months this corresponded with significantly greater medication adherence among those patients that remained in the study [55]. An intention to treat analysis, however, showed no significant intervention effect on medication adherence. Medication adherence was measured using an electronic pill container that recorded the time and frequency that the cover was opened. Analysis of psychological symptoms at the six month follow-up was inconclusive, with apparent improvements in symptom scores not replicated using an alternate method of analysis [54]. Randomisation occurred at the patient level, and neither pharmacists nor patients were blinded to their group allocation. A limitation of this method was that the same pharmacists provided services to both control and intervention patients. As the intervention studied was multifactorial, it was not clear whether the three face-to-face medication counselling sessions conducted by the pharmacists, or the "take-home" videos, were primarily responsible for changes in drug attitude, adherence and the symptoms scores observed.

### Services provided at medical centres and health maintenance organisations

Four studies reported patient education and treatment monitoring services for people prescribed antidepressant medications in the United States [56-59]. The patient education and treatment monitoring involved the pharmacists taking a medication history, providing information about the prescribed antidepressant medications, and conducting telephone and face-to-face follow-up. Two of the four studies, one controlled [56] and the other randomised controlled [57], were conducted at a staff model health maintenance organisation (HMO). Pharmacists' interventions in both studies were associated with significant improvements in adherence to antidepressant medications when calculated at the end of the six-month study periods. Medication adherence was calculated by reviewing prescription dispensing data, and reported using an intention-to-treat analysis. Both studies also demonstrated that involvement of the pharmacist was associated with a decrease in the number of visits to other primary care providers, although statistical significance was only achieved in one of the studies [56]. The other two studies were conducted at primary care medical practices. In one study over 16,000 consecutive patients attending nine practices were screened for depression using a self administered health survey [58]. Patients identified as having depression or dysthymia who agreed to participate in the study (n = 533) were randomised to intervention or control groups. Intervention patients were significantly more likely to be taking antidepressants at the six month follow-up. Additionally, patients who were taking their antidepressants at the six month follow-up had better depression symptom scores than those who had discontinued, but the overall symptom scores between intervention and control groups were not significantly different. In the other randomised controlled study, improvements in antidepressant adherence and depression symptom scores were similar in both intervention and control groups [59]. In this study antidepressant adherence was measured by asking patients how many days they took their antidepressant medication in the past month.

### Services provided at community mental health centres and outpatients' clinics

Three studies investigated the effect of pharmacist delivered services to community mental health centres and outpatients' clinics [60-62]. In a controlled trial, patients' case managed by a pharmacist working at a community mental health centre in the United States had significantly better personal adjustment scores than those receiving case management from a nurse, social worker or psychologist [60]. They were also significantly less likely to need help from other providers and rated themselves as more healthy. As part of the medication monitoring service provided, the pharmacist was allowed to adjust medication doses and dose timing, and prescribe or discontinue medications under supervision. Medication monitoring conducted by the pharmacist was estimated to cost 40% of equivalent medication monitoring conducted by the clinic psychiatrists when calculated on a per time basis. Although the pharmacist performed medication monitoring for more patients per month than the clinic psychiatrists, the pharmacist also spent longer per patient contact. This offset the overall cost savings of having a pharmacist perform the medication monitoring activities usually performed by a psychiatrist.

In a study of patients discharged home from hospital after admission for relapse of schizophrenia in Malaysia, those identified as having poor medication adherence were allocated to receive pharmacist medication counselling or standard care [61]. The importance of medication adherence was also reinforced by the patients' psychiatrists at follow-up visits, although it was not clear whether this applied only to intervention patients or both intervention and control patients. At the 12 month follow-up, patients who had been exposed to the intervention, and received a daily or twice daily medication treatment, had significantly fewer relapses that required hospitalisation than patients in the control group.

A study that evaluated the impact of providing mental health patients with a pharmacist generated medication care plan at the time of discharge found that patients with care plans were less likely to be readmitted to hospital than those without, however, this result was not statistically significant [62]. Information on the medication care plans included lists of discharge medications, a summary of the patient education that was provided, and the need to assess for specific potential adverse reactions. Community pharmacists who were provided copies of the care plans were also more likely to identify medication related problems for the discharged mental health patients than those pharmacists who were not provided copies of the care plans.

### Medication review in domiciliary and residential aged care settings

Components of medication review services provided by pharmacists include comprehensive medication history taking, patient home interviews, medication regimen review, and patient education [63]. Medication review studies described in the review were conducted for residents of aged care facilities or for those individuals living independently in the community identified to be at high risk of medication misadventure.

In a randomised controlled study of pharmacist conducted domiciliary medication reviews in the United States there were significant declines in the overall numbers and monthly cost of medications, but no significant difference in cognitive or affective functioning between the intervention and control groups [64]. This may have been due in part to the relatively short (6 week) follow-up period. The authors noted that many patients were unwilling to follow the pharmacist's recommendations to discontinue benzodiazepines and narcotic analgesics. A randomised controlled study of a pharmacist-led multidisciplinary initiative to optimise prescribing in 15 Swedish aged care facilities resulted in a significant decline in the use of antipsychotics, benzodiazepines and antidepressants by 19%, 37% and 59% respectively in the intervention facilities [65]. The study involved pharmacists participating in multidisciplinary team meetings with nurses, nurses' assistants and physicians at regular intervals throughout the 12-month study period. A follow-up study of the same intervention and control facilities three years later indicated the intervention facilities maintained significantly higher quality of drug use, with lower proportions of residents prescribed more than three drugs that could lead to confusion, non-recommended hypnotics and combinations of interacting drugs [66]. Neither study reported estimates of cost or clinical outcomes. A cluster randomised controlled study of a multidisciplinary primary care intervention at a HMO in the United States included a quarterly pharmacist medication review to address the potentially inappropriate use of medications commonly prescribed for mental illness. The researchers found the intervention had no impact on depression scores and the numbers of high risk medications prescribed at the 12 week follow-up [67].

Two additional cluster randomised controlled studies of pharmacists' medication reviews in residential aged care facilities demonstrated significant reductions in the number and cost of medications prescribed [68-70]. In one study 10.2% fewer residents were administered psychoactive medications and 21.3% fewer hypnotic medications [68]. The impact of medication reviews on mortality was measured in both studies, and a significant reduction was noted in one [70]. Despite the significant reduction in mortality, patients in the intervention facilities experienced a greater deterioration in cognitive function and behavioural disturbance than those in the control facilities.

### Educational visiting to general medical practitioners

In the Netherlands, pharmacotherapy meetings to optimise prescribing are undertaken as part of routine clinical practice by groups of local community pharmacists and general medical practitioners. A cluster randomised controlled trial of inter-professional (pharmacotherapy) meetings to discuss prescribing of antidepressant medications resulted in a significant reduction in the prescribing of highly anticholinergic antidepressants to elderly people by 40% compared to a control group of practitioners that did not receive the prescribing support [71]. In comparison, educational visiting (academic detailing), reduced prescribing of highly anticholinergic antidepressants by 30%.

Four additional studies evaluated the impact of pharmacists' educational visits to general medical practitioners to optimise the prescribing of benzodiazepines and other psychotropic medications commonly prescribed for mental illness [72-75]. The two papers that reported health professional satisfaction indicated that the educational visits were acceptable and well received [73,74]. In a controlled trial, two types of pharmacists' educational interventions (a one-on-one presentation to prescribers with individualised feedback and a group presentation to prescribers about the use of benzodiazepines) did not produce significant changes to the prescribing of benzodiazepines at a prepaid group practice in the United States when compared to a control group that did not receive an educational intervention [72]. An Australian cluster randomised controlled study of a pharmacist's educational visits to general medical practitioners providing services to residential care facilities detected no significant differences in the use of psychotropic medications between intervention and control facilities. The only exception was a significant increase in the use of "as required" antipsychotic medications in the intervention facilities [73]. This differed from results of an earlier cluster randomised controlled study in the United States that found that educational visits by a pharmacist were associated with a significant decline in prescribing of potentially inappropriate psychotropic medications in intervention facilities [75]. Another Australian study of educational visits to general medical practitioners, conducted by three physicians and one pharmacist, reported a significant reduction in the prescribing of benzodiazepines in both intervention and control groups, but the difference between groups was not significant [74]. The authors accounted for this overall reduction by a corresponding decline in the rate of diagnoses of anxiety and insomnia, and the possible awareness of prescribing related issues generated by asking general medical practitioners to conduct a self-audit of their prescribing.

## Discussion

Given the extent of mental illness in the community and in aged care, and the fact people with mental illness frequently report concerns about their prescribed medications, services directed toward optimising the use of medications for mental illness fulfil an important public health need. As evidenced by the large number of papers excluded from this review, many studies of community pharmacy services to optimise the use of medications for mental illness have been descriptive, lacked parallel control groups or have been qualitative in nature. The controlled studies included in this review provide some evidence of the potential value of including pharmacists in mental health care across a range of settings and patient populations.

Studies included in the review utilised a range of randomisation techniques, however, the review did not attempt to characterise the quality of the randomisation beyond whether randomisation occurred at the patient, practice or residential aged care facility level. The majority of the studies involved less than five pharmacists, and 10 out of the 22 papers described interventions where just one pharmacist was involved. Studies involving small numbers of pharmacists may have good internal consistency, but the results obtained may not be generalisable to outcomes of services provided by the wider pharmacy profession. In several studies the pharmacists' interventions were components of multidisciplinary team approaches to improving mental health care. The challenge of evaluating complex and multi-factorial interventions, which by their nature depend on the context in which the intervention takes place, has been described [76].

Five studies assessed the impact of pharmacists' provision of medicines information and treatment monitoring for people commencing antidepressant therapy. Three of the five studies demonstrated that involvement of the pharmacist was associated with a significant improvement in medication adherence and/or medication use rates when measured using an intention to treat analysis. One further study demonstrated significant improvements in medication adherence among patients who received three pharmacist counselling sessions; however, this was not significant when measured using an intention to treat analysis. Given the high rates of antidepressant discontinuation during the first three months of treatment, pharmacists have a potentially important role in providing medicines information and conducting treatment monitoring for those patients at high risk of medication non-adherence. No studies of pharmacists' treatment monitoring for people commencing antidepressant therapy compared monitoring provided by pharmacists to monitoring conducted by other health professionals. A separate study of antidepressant treatment monitoring conducted by nurses also demonstrated improved medication adherence [77].

Despite people with psychotic disorders having reported unmet medicines information needs, relatively few controlled studies assessed community pharmacy services for users of antipsychotic medications. Other studies have suggested that service provision by pharmacists may be limited by not having access to patients' medical histories [46], a lack of specific training to counsel this patient population [46], and pharmacists' attitudes towards people with mental illness [48]. Further well designed research into community pharmacy services for users of antipsychotic medications is needed before conclusions can be made about the potential of such services to reduce hospital readmission and the cost of health care.

Pharmacist conducted medication management reviews appear a valuable strategy to identify potential medication related problems among people taking medications for a mental illness. The included studies demonstrated that such reviews can reduce the numbers of potentially inappropriate psychotropic medications used for mental illness prescribed to elderly people in residential aged care settings. Only one study made the link between a reduction in psychotropic medication use and improved adherence to national prescribing guidelines [66]. The value of pharmacist conducted medication reviews for people with mental illness may not be limited to optimising the use of mental health medications. Physical health care for people with mental illnesses is often less than optimal, and pharmacist conducted medication reviews may be a comprehensive strategy to improve medication use for both mental and physical illnesses. The tendency among health professionals to focus solely on the management of the mental illness among people with both mental and physical illnesses has been described in the literature [78].

Educational visiting has been shown to modify prescribing behaviour [79]. The reviewed studies reported pharmacists' interventions that were well received by prescribers, but produced differing results as to whether such visits were associated with changes in prescribing behaviour. This may have been because efforts to reduce prescribing of potentially inappropriate medications were not accompanied by information about alternate treatments, or because patients were reluctant to discontinue taking benzodiazepine medications. In the Dutch study that did produce a significant impact on prescribing patterns, information about the problems associated with prescribing highly anticholinergic antidepressants was accompanied by information about prescribing more appropriate antidepressant medications [71]. Additionally, pharmacists' initiatives to improve prescribing may be most effective when both the pharmacists and general medical practitioners have an opportunity to build rapport. The practitioners involved in the Dutch study were those routinely involved in providing care to the patient populations discussed. Data presented on prescribing at these meetings were relevant and specific to the local area in which the meetings took place.

## Conclusion

The review of the international literature highlights the range of pharmaceutical services provided by community pharmacists in Australia that are potentially well suited to assisting patients and prescribers optimise the use of medications for mental illness. These data show that medication counselling and treatment monitoring conducted by pharmacists can improve medication adherence among people commencing antidepressant therapy. Pharmacist conducted medication reviews and resulting recommendations to optimise medication regimens may reduce the numbers of potentially inappropriate medications for mental illness prescribed to elderly people. This review of the available published evidence supports the continued expansion of pharmaceutical service delivery to people with mental illness, but identified the need for further well-designed research in specific areas. Future studies are needed to assess the cost-effectiveness and clinical implications of pharmacists working as members of multidisciplinary community mental health teams, and as providers of pharmaceutical services to people with psychotic disorders.

## Authors' contributions

SB conducted the literature search and wrote the manuscript. AJM assisted in the literature search and in the writing of the manuscript. PA proof read drafts of the manuscript. PW and TFC participated in the conceptualisation of the review and assisted in the writing of the manuscript.
